# A Survey of Trunk Disease Pathogens within Citrus Trees in Iran

**DOI:** 10.3390/plants9060754

**Published:** 2020-06-16

**Authors:** Nahid Esparham, Hamid Mohammadi, David Gramaje

**Affiliations:** 1 Department of Plant Protection, Faculty of Agriculture, Shahid Bahonar University of Kerman, Kerman 7616914111, Iran; hmho279@yahoo.com; 2 Instituto de Ciencias de la Vid y del Vino (ICVV), Consejo Superior de Investigaciones Científicas, Universidad de la Rioja, Gobierno de La Rioja, 26007 Logroño, Spain

**Keywords:** bscogniauxia, botryosphaeriaceae, cadophora, citrus dieback, colletotrichum, phaeoacremonium

## Abstract

Citrus trees with cankers and dieback symptoms were observed in Bushehr (Bushehr province, Iran). Isolations were made from diseased cankers and branches. Recovered fungal isolates were identified using cultural and morphological characteristics, as well as comparisons of DNA sequence data of the nuclear ribosomal DNA-internal transcribed spacer region, translation elongation factor *1α*, β-tubulin, and actin gene regions. *Dothiorella* *viticola*, *Lasiodiplodia theobromae*, *Neoscytalidium* *hyalinum*, *Phaeoacremonium (P.) parasiticum*, *P. italicum*, *P. iranianum*, *P. rubrigenum*, *P. minimum*, *P. croatiense*, *P. fraxinopensylvanicum*, *Phaeoacremonium* sp., *Cadophora luteo-olivacea*, *Biscogniauxia* (*B*.) *mediterranea*, *Colletotrichum gloeosporioides, C. boninense*, *Peyronellaea* (*Pa*.) *pinodella, Stilbocrea* (*S*.) *walteri*, and several isolates of *Phoma*, *Pestalotiopsis*, and *Fusarium* species were obtained from diseased trees. The pathogenicity tests were conducted by artificial inoculation of excised shoots of healthy acid lime trees (*Citrus aurantifolia*) under controlled conditions. *Lasiodiplodia theobromae* was the most virulent and caused the longest lesions within 40 days of inoculation. According to literature reviews, this is the first report of *L. theobromae* and *N. hyalinum* on citrus in Iran. Additionally, we report several *Phaeoacremonium* species, *S. walteri*, *Pa. pinodella* and *C. luteo-olivacea* on citrus trees for the first time in the world.

## 1. Introduction

Iran is the sixth largest Citrus producer, accounting for 3.3% of the world’s Citrus production, which yielded 4.1 million tons in 2016 [1]. A total of 276,000 ha of various *Citrus* species are cultivated in Iran, including sweet orange (*Citrus sinensis* L.), acid lime (*C. aurantifolia* (Christm.) Swingle), sour orange (*C. aurantium* L.), mandarin (*C. reticulata* Blanco), lemon (*C. limon* (L.) Osbeck), and grapefruit (*C. paradisi* Macfad). The most important producing regions in Iran are Mazandaran, Fars, Hormozgan, Giroft, and Kahnouj. 

Fungal trunk diseases have been studied in detail in grapevine, which are the main biotic factor limiting vineyard productivity and longevity [2]. However, recent findings of high incidence in stone and pome fruits, small fruits, nut crops, citrus, and olive worldwide highlight the need for a focus on this novel group of hosts [3]. Trunk diseases are caused by a broad range of taxonomically unrelated fungi that primarily infect wood hosts through winter pruning wounds, thus colonizing the vascular tissues. Members of the families Botryosphaeriaceae, Togninaceae, Diatrypaceae, Diaporthaceae, as well as several basidiomycetes are included in this group of fungi. Members of Diatrypaceae (Xylariales) can often be observed on dead wood and bark of a wide range of plant species around the world. Nevertheless, some species of this family are reported as putative plant pathogens on fruit, ornamental, and forest trees [4,5,6,7,8]. Some species of *Eutypella* have been previously isolated from citrus species, including *Citrus limon*, *C. paradisi*, *C. maxima*, and *C. aurantium* in Australia, Argentina, Brazil, Coted’Ivoire, Philippines, and USA [9,10,11,12,13,14]. Diatrypaceae spp. were also isolated from citrus trees in Australia [15].

Species of Botryosphaeriaceae have a cosmopolitan distribution and have been associated with numerous plant species worldwide [16,17,18]. Many species of the genera *Lasiodiplodia* [19,20,21,22,23], *Diplodia*, *Dothiorella, Neofusicoccum* [20], and *Neoscytalidium* [14,20] have been previously reported to affect citrus trees. Togniniaceae (Togniniales), with the well-known asexual morph genus *Phaeoacremonium*, is another family of fungi traditionally associated with dieback, canker, and yellowing of various fruit, forest, and ornamental trees, worldwide [24,25,26,27,28,29,30,31]. To date, 56 *Phaeoacremonium* species have been identified from woody hosts [32]. The most prevalent *Phaeoacremonium* species isolated from woody hosts are *P. minimum*, followed by *P. parasiticum* [33]. Dieback and related disease symptoms have been achieved by inoculating *Phaeoacremonium* species onto several hosts such as *Prunus* spp., kiwifruit, oak, and grapevine [33,34]. *Phaeoacremonium* species isolated from grapevine have been intensively studied because of their involvement in two trunk diseases, Petri disease in young vines and esca in mature vines [32,35]. To our knowledge, there are no reports of *Phaeoacremonium* species affecting *Citrus* spp.

In spring 2014, a severe decline of citrus trees was noticed in some orchards in Bushehr (Bushehr province, Iran). External disease symptoms included chlorosis of leaves, defoliation, branch and shoot cankers, and dieback. Internal wood symptoms ranged from brown to black wood streaking and black spots to wedge-shaped necrosis, irregular wood discoloration, central necrosis, and arch-shaped necrosis. Many fungi associated with trunk diseases have been isolated from several woody hosts in Iran, including grapevine [36,37], pome and stone fruit trees [28,38], and ornamental and forest trees [29,31,39,40]. However, little information is presently available on the causal agents of the severe decline of citrus trees in Iran. Therefore, the aim of this study was to investigate the etiology of fungal trunk diseases associated with wood necrosis of citrus trees in Iran and to determine their pathogenicity.

## 2. Results

### 2.1. Field Survey and Diversity of Disease Symptoms

**Figure 1:**
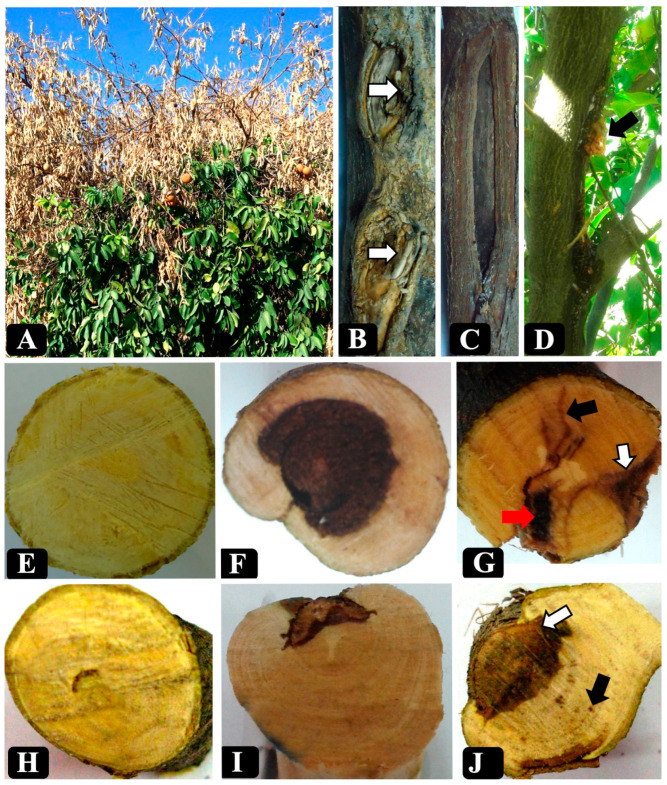
Diversity of external (**A**–**D**) and internal (**E**–**I**) trunk disease symptoms on Citrus species in Iran (**A**) a severe dieback on *Citrus sinensis*; (**B**) two cankers on a trunk of a *C. sinensis* tree indicated by arrows; (**C**) an extended canker on the branch of *Citrus aurantifolia* indicated by arrow; (**D**) gummosis on *Citrus limetta*; (**E**) cross-section of a healthy branch of *C. aurantifolia*; (**F**) central necrosis on *C. sinensis*; (**G**) Co-occurrence of brown wood streaking (black arrow), wedge-shaped necrosis (white arrow) and irregular wood necrosis (red arrow) on *C. sinensis*; (**H**) Arch-shaped necrosis on *C. aurantifolia*; (**I**) a young wedge shaped necrosis on *Citrus reticulata*; (**J**) Co-occurrence of wedge-shaped necrosis is indicated by the white arrow and black spots are indicated by the black arrow on the *C. sinensis*.

In this study, wood samples were collected from lime (46 trees), sweet lemon (23 trees), sweet orange (22 trees), mandarin (eight trees), sour orange (four trees) and lemon (three trees). Citrus trees showed various external disease symptoms, including yellowing, canker, defoliation, dieback, cracking of the bark associated with gumming, and sooty cankers (form a black powder underneath the bark). Examination of infected branches from symptomatic trees revealed different types of wood discoloration in cross-sections, black to brown streaking in the wood, wedge-shaped necrosis, black spots, irregular wood discoloration, central necrosis, and arch-shaped necrosis (Figure 1).

### 2.2. Fungal Isolation and Morphological Identification

In this survey, 326 fungal isolates were collected from citrus trees (Table 1). According to colony appearance, culture characteristics, and microscopic structures, the main fungal isolates were classified as *Phaeoacremonium* spp., Botryosphaeriaceae spp. *Cadophora* sp., *Colletotrichum* spp., Peyronellaea sp., *Phoma* spp., and *Biscogniauxia mediterranea*. Thirty-nine isolates (11.96% of total isolates) were identified as *Phaeoacremonium* species and characterized by beige to medium brown flat slow-growing cultures on potato dextrose agar (PDA; Merck, Darmstadt, Germany) and on malt extract agar (MEA; 2% malt extract, Merck, Darmstadt, Germany). Septate hyphae were single or fasciculate, and three types of phialides, variable in shape and size (I, II, and III types), were recorded in these isolates [41]. Morphological features of 49 isolates (15.03%) were consistent with the description of species of Botryosphaeriaceae [16,17,42]. These isolates were characterized by dark green to gray or fast-growing gray mycelium on the PDA. All isolates produced fruit bodies, pycnidia, on pine needles within 15–35 days. Conidia were pigmented or hyaline. These isolates belonged to the genera *Lasiodiplodia*, *Neoscytalidium*, and *Dothiorella*. Twelve isolates of the phialides fungus were identified as *Cadophora* sp. These isolates formed flat, felty, and black-olivaceous and white to gray colonies on PDA, and their conidia were ellipsoid or elongate. Cultural and morphological characteristics observed were similar with the description of the *Cadophora* spp. [43,44]. Based on morphological characteristics, the remaining isolates were classified to *Colletotrichum*, *Peyronellea*, *Pestalotiopsis*, *Fusarium*, *Microsphaeropsis*, *Alternaria*, *Trichoderma*, *Paecilomyces*, *Aspergillus*, *Penicillium*, *Phoma*, *Biscogniauxia*, and *Stilbocrea* genera.

No association was found between wood symptoms and fungal species. Dual infections by trunk disease fungi in a single tree occurred. *Phaeoacremonium parasiticum* and *P. italicum* were isolated from one tree of *C. aurantifolia*; *P. parasiticum*, *P. croatiense*, and *Do. viticola* from one tree of *C. sinensis*, *L. theobromae* and *Neoscytalidium hyalinum* from one tree of *C. aurantifolia*, and *C. luteo-olivacea* and *P. croatiense* from one tree of *C. limetta*. In addition, some fungal species grew from an individual wood segment, such as *Stilbocrea walteri* and *P. fraxinopennsylvanicum* from *C. limon*.

### 2.3. Molecular Characterization and Phylogenetic Analyses

BLASTn searches in GenBank showed that the nuclear ribosomal DNA-internal transcribed spacer region (ITS) and translation elongation factor *1α* (tef1-α) sequences of Botryosphaeriaceae isolates had 99–100% identity with isolates of *Lasiodiplodia theobromae* (strain CBS559.70), *Neoscytalidium hyalinum* (strain CBS 145.78), and *Dothiorella viticola* (strain CBS 117006). The ITS sequences of the *Cadophora* isolates had 99–100 % identity with isolates previously identified as *Cadophora luteo-olivacea* in GenBank (strain CBS 855.69). ITS and β-tubulin (BT) sequences of *Colletotrichum* isolates were identical to isolates previously reported as *Colletotrichum gloeosporioides* (ITS: strain CBS 132465; BT: strain CBS 100471) and *Colletotrichum boninense* (ITS and BT: strain CBS:123755) in GenBank. ITS and BT sequences of *Peyronellea* isolates in our study showed 99–100 % identity with those isolates previously submitted as *Didymella pinodella* (strain CBS 531.66) in GenBank. ITS and tef1-α sequences of *Stilbocrea* isolates from citrus trees had 99–100% identity with *Stilbocrea walteri* (strain NQI). Regarding the *Biscogniauxia* isolates, ITS of our isolates had 99–100% identity with isolates previously identified as *Biscogniauxia mediterranea* (strain CBS 129072).

**Figure 2:**
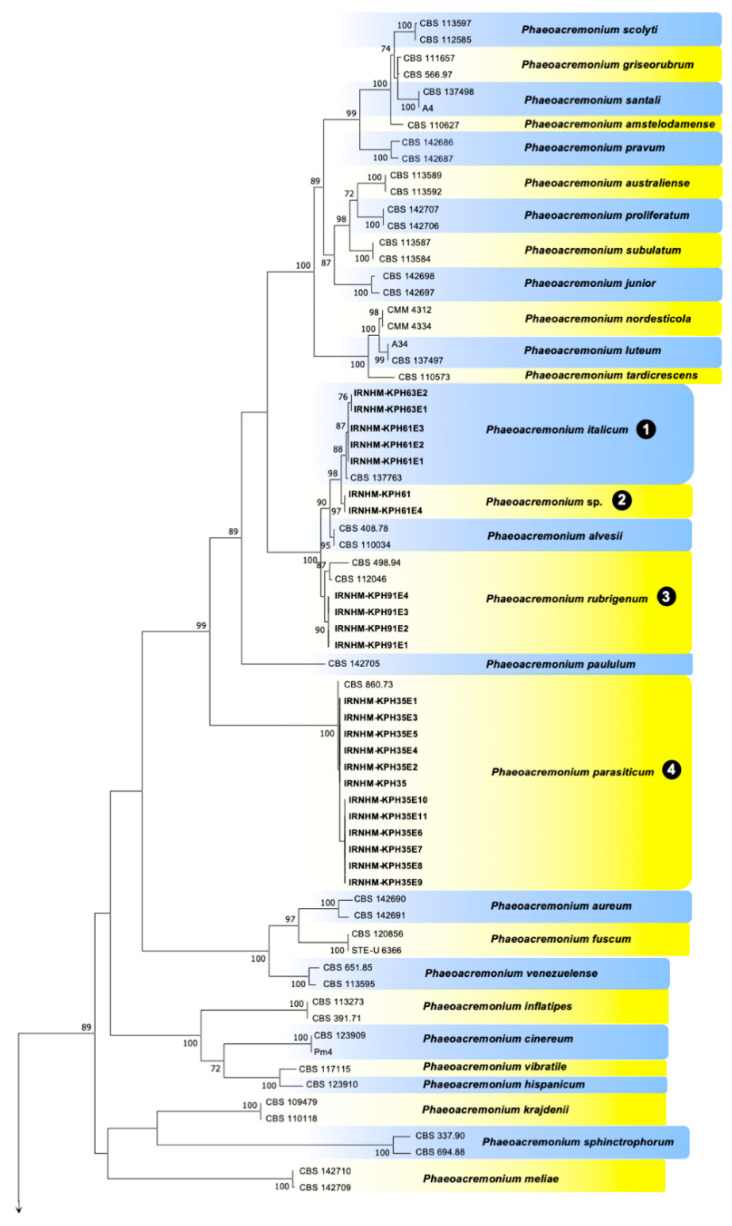

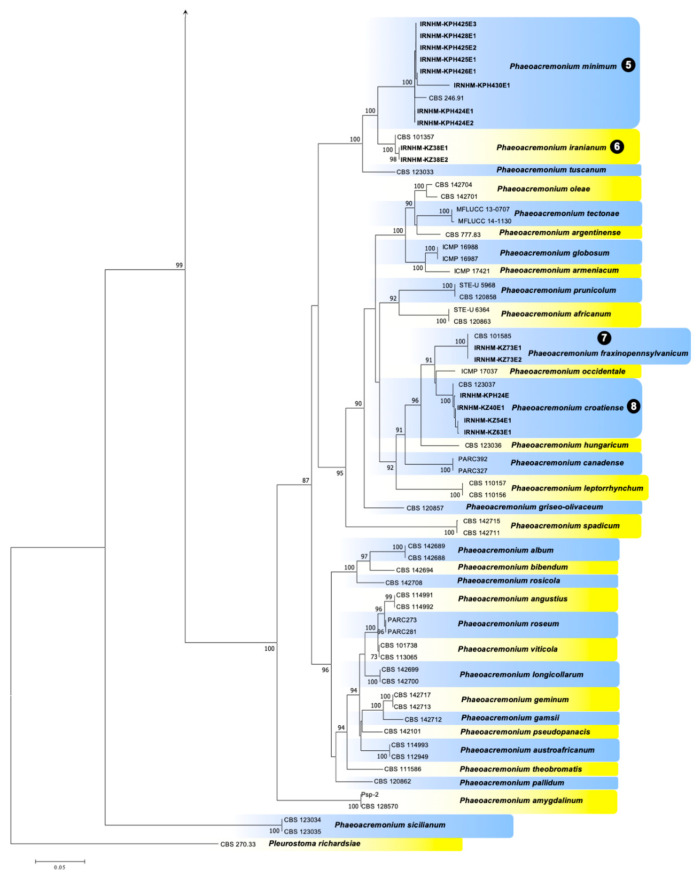
Maximum likelihood phylogeny of *Phaeoacremonium* spp. according to concatenated alignments of the actin (ACT) and beta-tubulin (BT) gene regions. Support values less than 70% bootstrap were omitted. Maximum likelihood bootstrap percentages are indicated at the nodes. Isolates obtained in this study are indicated in bold. The eight clades associated with the *Phaeoacremonium* spp. obtained in this study are indicated by numbers.

Datasets of the BT and actin (ACT) alignments of *Phaeoacremonium* were congruent and could be combined (*p* = 0.225). The Hasegawa–Kishino–Yano model (HKY) with gamma distributed with invariant sites rates (G+I) was identified as the BIC best-fit nucleotide substitution model by the jModelTest for the *Phaeoacremonium* multi-locus analysis. Maximum likelihood (ML) of the combined ACT-BT regions provided a phylogeny with 98 to 100% ML bootstrap support for all species-level clades, with the exception of *P. alvesii* (paraphyletic, 87% bootstrap support), *P. griseorubrum* (paraphyletic, 66% bootstrap support), *P. roseum* (89% bootstrap support), and *P. viticola* (paraphyletic with regard to *P. roseum* and *P. angustius*) (Figure 2). The 39 strains from Iran clustered in eight clades (*P. italicum*, *Phaeoacremonium* sp., *P. rubrigenum*, P. parasiticum, *P. minimum*, *P. iranianum*, *P. fraxynopennsylvanicum*, and *P. croatiense*). The isolates of the clade 2 grouped together in a polyphyletic clade with 100% bootstrap support with the *P. italicum* as a closely related species. The BT and ACT sequences of the second clade of *Phaeoacremonium* isolates were 98% (BT) and 98.77% (ACT) identical to those of *P. italicum* CBS 137763 (GenBank KJ534074, KJ534046). Three nucleotides varied in the ACT region and ten nucleotides in the BT region between the second clade of *Phaeoacremonium* isolates and the *P. italicum* CBS 137763 sequences.

### 2.4. Pathogenicity Test

**Figure 3:**
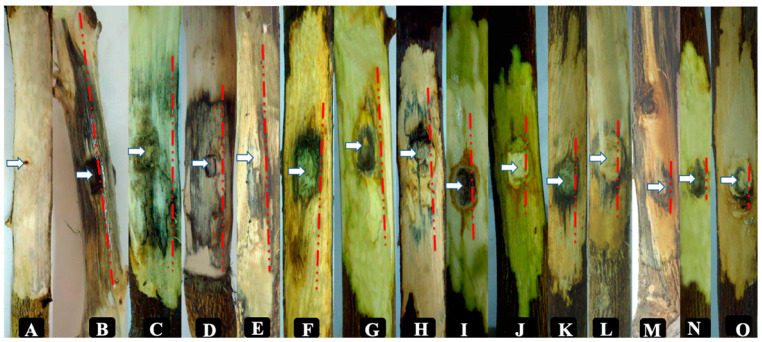
Pathogenicity tests of fungal species inoculated onto Citrus aurantifolia detached shoots, 40 days after inoculation: (**A**) control; (**B**,**C**) Lasiodiplodia theobromae; (**D**); Dothiorella viticola; (**E**) Neoscytalidium hyalinum; (**F**,**G**) Phaeoacremonium parasiticum; (**H**) Cadophora luteo-olivacea; (**I**) Phaeoaremonium minimum; (**J**) Phaeoacremonium iranianum; (**K**) Colletotrichum gloeosporoides; (**L**) Phaeoacremonium fraxinopennsylvanicum; (**M**) Phaeoacremonium italicum; (**N**) Stilbocrea walteri; (**O**) Phaeoacremonium rubrigenum; (white arrows show the point of inoculation, and the red dashed lines indicate the lesion length caused by each isolate).

**Figure 4:**
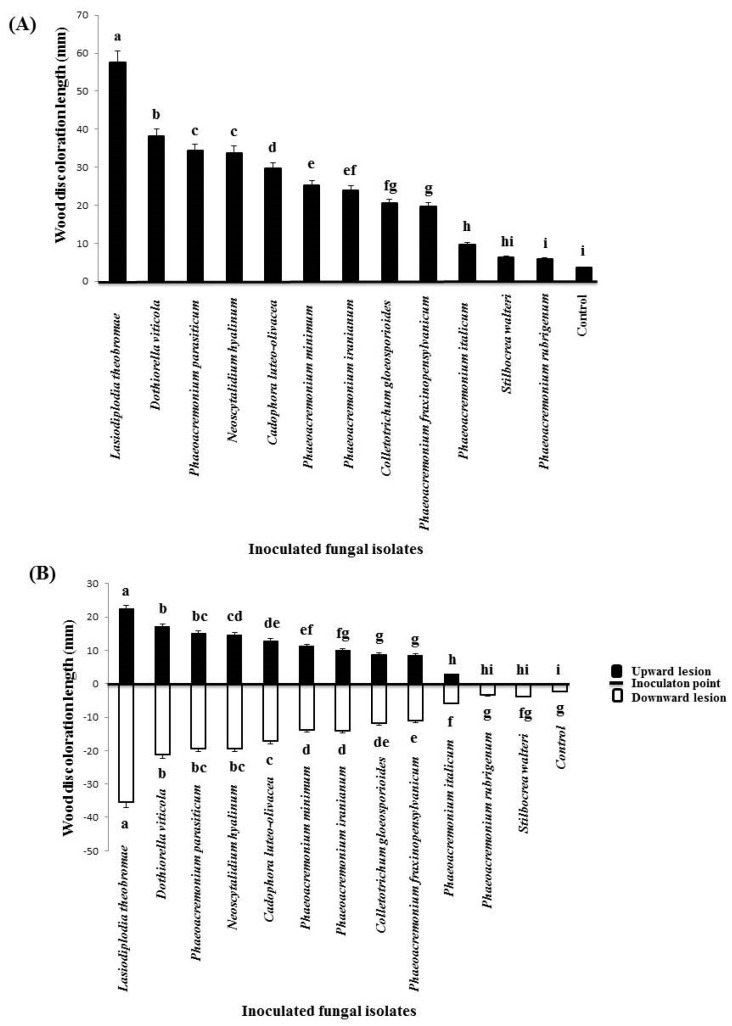
Total (**A**) and upward and downward (**B**) wood lesion size produced by inoculated isolates onto *Citrus aurantifolia* detached shoots, 40 days after inoculation. Different letters in boldface indicate significant differences at *p* = 0.05. Bars represent standard error of the means.

Mean lengths of wood discolorations caused by inoculated isolates obtained from Citrus species on the detached shoots of *C. aurantifolia* are shown in Figure 3 and Figure 4. Our results showed a variation in the total (Figure 4a), and both the upward and downward lesion lengths (Figure 4b) from the point of inoculation and re-isolation frequencies of inoculated isolates on lime shoots. *L. theobromae* was the most aggressive fungal species and produced the longest necrotic lesions (57.67 mm) on the inoculated shoots followed by *Do. viticola* (38.17 mm) and *P. parasiticum* (34.33 mm) (Figure 4a). In contrast, two species of *S. walteri* (6.33 mm) and *P. rubrigenum* (6.00 mm) produced the smallest wood lesions on the inoculated shoots, and no significant differences were observed between these species and the control treatments (3.67 mm).

All inoculated fungi caused longer basipetal than acropetal lesions on the lime shoots (Figure 4b). Of the isolates inoculated, 10 species caused downward and upward wood lesions that were significantly different to those in the control (*p* < 0.05). *L. theobromae* also produced the longest wood lesion lengths both in upward (22.34 mm) and in downward (35.33) directions, while *S. walteri* (upward = 2.5, downward = 3.83 mm) and *P. rubrigenum* (upward = 2.5, downward = 3.50 mm) did not cause any significant necrotic lesion lengths both in the downward and in the upward directions compared to the control treatments (upward = 1.34, downward = 2.33 mm) on the inoculated shoots. Re-isolation percentages were between 40.0% (*C. luteo-olivacea*) and 100% (*L. theobromae* and *N. hyalinum*) on the inoculated lime shoots, and no fungal isolates were recovered from control treatments.

## 3. Discussion

This study shows the high incidence and severity of fungal trunk pathogens associated with wood decay symptoms of six *Citrus* species (*C. sinensis*, *C. aurantifolia*, *C. reticulate*, *C. limetta*, *C. aurntium*, and *C. limon*) in Iran. During the last decade, extensive studies have been done on fungal trunk pathogens of fruit trees, including grapevine [36,37], stone [38], and pome fruit trees [28,30], pistachio [45], almond [46,47,48], walnut [49,50], pomegranate, and fig trees [51] in Iran. The current study shows that *Citrus* also represents a rich catch host for fungi associated with trunk diseases in this country. Different trunk disease fungi often co-occurred in the same tree and even in the same type of symptom, thus showing the complexity of the etiology of wood symptoms observed. The co-infection of several trunk disease fungi on woody crops could lead to an increase in disease severity compared to the single occurrence of a fungal species, as it has been previously demonstrated on grapevine with Botryosphaeriaceae and *Ilyonectria* spp. [52].

Morphological comparisons of trunk disease fungi often reveal an overlap between species in several characters [13,16,41]. In our study, the use of these characters to distinguish fungal species within a genus or family was inadequate, thus highlighting the convenience of DNA-based methods for such purposes. This is particularly important for species of the genus *Phaeoacremonium* [41]. Throughout this survey, seven *Phaeoacremonium* species, including *P. parasiticum*, *P. minimum*, *P. rubrigenum*, *P. italicum*, *P. iranianum*, *P. croatiense*, *P. fraxinopennsylvanicum*, and an unidentified species of *Phaeoacremonium* were recovered from *Citrus* spp. showing a decline in symptoms. All *Phaeoacremonium* species reported herein have been found associated with grapevine [41,53,54,55]. *P. parasiticum* was the dominant *Phaeoacremonium* species in this study, with 12 isolates collected from *C. sinensis* and *C. aurantifolia*. This fungus has previously been reported from *C. reticulata* in Iran [56], and from various fruit trees, such as grapevine [41], *Actinidia chinensis* [57], *Cydonia oblonga*, *Ficus carica* [58], *Olea europaea* [58,59], *Malus domestica* [28,58], *Prunus armeniaca* [24], *Prunus avium* [60], *Punica granatum* [58], and *Pyrus communis* [28] worlwide. In our study, *P. minimum, P. rubrigenum* and *P. italicum* were isolated only from *C. aurantifolia*. Similar to *P. parasiticum, P. minimum* was also reported from a wide range of fruit trees, including *A. chinensis* [61], *A. deliciosa* [62], *C. oblonga* [28,58], *M. domestica* [25,28,63], *O. europaea* [64], *P. armeniaca* [24], *Prunus dulcis* [57], *P. pennsylvanica* [65], *Prunus salicina* [24], *P. granatum* [58], *P. communis* [25,28], in Iran and other parts of the world. *P. rubrigenum* has previously been reported from *C. oblonga* [28], *O. europaea* [59], and *P. communis* [28]. More recently, fruit tree infections by *P. italicum* have also been reported from South Africa and this fungus has been isolated from *C. oblonga*, *Ficus carica*, *M. domestica*, *O. europaea*, *P. persica*, and *P. granatum* in this country [58]. *P. croatiense* was isolated from *C. sinensis* and *C. limetta*, while *P. fraxinopennsylvanicum* was isolated form *C. aurantium*. Related to fruit trees, *P. fraxinopennsylvanicum* was previously reported to affect *A. deliciosa* [62], *M. domestica* [28,58], *P. salicina* [24], and *Pyrus communis* [25], while *P. croatiense* was only reported from grapevine [54]. Our research confirms the broad distribution of *Phaeoacremonium* spp. affecting woody crops, and provides their first record on citrus trees in the world.

Three species of Botryosphaeriaceae, namely *N. hyalinum*, *Do. viticola* and *L. theobromae* were obtained from citrus trees in this study. *Neoscytalidium hyalinum* was isolated from *C. aurantifolia* and *C. limetta*, *Do. viticola* was recovered from *C. sinensis*, *C. aurantifolia*, and *C. aurantium*, and *L. theobromae* was associated with *C. sinensis* and *C. aurantifolia*. Several species of Botryosphaeriaceae are known to dieback and branch cankers in *Citrus* spp. worldwide [14,20,22,23,66,67,68,69,70]. *Dothiorella viticola* has been previously reported to cause gummosis in citrus in California [20] and Tunisia [71]. This fungus has also been reported from cultivar Parent Washington on sour orange rootstock [68], *C. sinensis* and *C. latifolia* Tan. in California [20], and *C. sinensis* in New Zealand [72]. Abdollahzadeh et al. reported this species from *Citrus* sp. in Guilan province of Iran [73]. Our study provides the first report of this fungus from *C. aurantifolia* and *C. aurantium*.

*Neoscytalidium hyalinum* has been reported as the most prevalent Botryosphaeriaceae species associated with citrus branch cankers in the desert regions of southern California [14]. This fungus has been recovered from *C. paradise* showing gummosis in California [20] and also from *C. sinensis* in Italy [74]. Therefore, our work is the first report of *N. hyalinum* from two *Citrus* species, *C. aurantifolia* and *C. limetta*. *L*. *theobromae* has been previously reported from some *Citrus* species, including *C. limon* in Chile [23] and Persian lime (*Citrus latifolia*) trees in Mexico [70]. Our study represents the first report of this species on *C. sinensis* and *C. aurantifolia*. 

In the current study, 12 isolates of *Cadophora luteo-olivacea* were obtained from *C. reticulata* and *C. limetta. C. luteo-olivacea* has previously been reported with black vascular streaking and a decline in the symptoms characteristic of Petri disease on grapevine [44,54,75,76], bark cracks of kiwifruit [62], and from pear fruits showing dark-brown and slightly sunken spots [77]. Aside from these reports, little is known regarding the role of *Cadophora* species involved in trunk diseases of trees. This is the first time that *C. luteo-olivacea* has been found on *Citrus* spp.

Most species of the genus *Biscogniauxia* are reported from forest trees, mainly from *Quercus* spp. [78,79,80]. Some species of this genus have also been found associated with fruit trees such as *B. pruni* and *B. granmoi* on *Prunus padus* [81,82], *B. marginata* on *M. communis* [83], *B. rosacearum* on *P. communis*, *C. oblonga* and *Prunus domestica* [84], and *B. capnodes* on *Averrhoa carambola* [85]. *Biscogniauxia mediterranea* is known to be the causal agent of charcoal cankers on a wide range of trees worldwide, in particular *Quercus* spp. [79,80,86]. In Iran, this pathogen was already reported from *C. sinensis* [87], along with other woody hosts, such as *Quercus castaneifolia* [88], *Zelkova carpinifolia* [89], *Q. brantii* [89], and *Amygdalus scoparia* [90].

In the current study, eight isolates of *Stilbocrea walteri* were isolated from *C. aurantifolia, C. aurntium*, and *C. limon*. This species was originally reported from dead corticated branches of *Quercus ilex* in Portugal [91], and to our knowledge, it has not been reported from necrotic wood tissues of trees. Therefore, this study is the first report of this species in Iran and on *Citrus* species worldwide.

*Peyronellea pinodella* (Didymellaceae) is a destructive necrotrophic pathogen on some plant families, including *Fabaceae*, *Amaranthaceae*, *Asteraceae*, *Amaryllidaceae*, *Appiaceae Rubiaceae*, *Malvaceae*, *Poaceae*, and *Polemoniaceae* [92]. To date, there is no report on the occurrence of *P. pinodella* on *Citrus* species and this is the first data on the occurrence of this species on *C. sinensis* and *C. aurantifolia*.

Two species of *Colletotrichum* were found to be associated with trunk diseases of citrus trees in this work, *C*. *gleoesporioides* on *C. sinensis* and *C. limetta* and *C. boninense* on *C. limetta*. Several species of *Colletotrichum* are associated with fruit and leaf anthracnose diseases of *Citrus* species; however, other diseases such as twig and shoot dieback caused by *Colletotrichum* spp. have been documented on citrus trees [14,93]. *Colletotrichum gloeosporioides* has been reported from a wide range of fruit trees such as strawberry, olive, almond, mango, apple, avocado, and citrus [94]. This fungus was found to be associated with twig dieback of lemon trees in Portugal [93]. *C. boninense* has been associated with fruit and leaf anthracnose on citrus trees [95,96]. According to a recent study, some *Colletotrichum* species have been isolated and reported from stems of citrus trees in Iran. These included *C. karstii* from *C. aurantifolia* and *C. sinensis* and four species, *C. gloeosporioides*, *C. novae–zelandiae*, *C. siamense*, and *C. fructicola* from *C. sinensis* [97]. Therefore, our study represents the first report of *C*. *gleoesporioides* and *C. boninense* from branches of *C. limetta*.

In our work, six isolates of *M. olivacea* were obtained from sweet orange. This fungus has been reported from various plant species worldwide. This taxon has previously been isolated as an endophytic species from *P. persica* [98], from xylem and stems of *Pinus sylvestris* [99] and Chilean gymnosperms [100]. Carlucci et al. isolated this species from internal wood discoloration of olive trees in Italy [101]. *Microsphaeropsis olivacea* has also been isolated and reported from some woody plants, such as *Prunus cerasus, P. avium* [102], and Persian oak (*Quercus brantii*) [103] in Iran. To our knowledge, this is the first report of *M. olivacea* on citrus trees. Several isolates of *Fusarium*, *Pestalotiopsis*, *Phoma, Penicillium, Aspergillus, Trichoderma*, and *Alternaria* species were also obtained from *Citrus* species in this study. Therefore, more studies are needed on these taxa in order to elucidate their potential impact on citrus trunk diseases.

Pathogenicity of selected fungal species in detached shoots of lime tree were confirmed in the current study. Results revealed that *L. theobromae* was more virulent on lime shoots than other species. In contrast to our results, Bautista-Cruz et al. reported that *L. theobromae* was the least virulent species when inoculated in Persian lime branches [70]. Several factors differed from the study carried out by Bautista-Cruz et al. and might have contributed to the discrepancy between the experiments, including the type of planting material inoculated, the environmental conditions for disease development, the time for virulence assessment, and the fungal strain used in the pathogenicity test. *L. theobromae* has been considered the most aggressive species on *Eucalyptus* [104,105], grapevine [42,106], and pistachio trees [107]. *Lasiodiplodia theobromae* was considered an important pathogen on greengage, sour cherry, peach, apricot, cherry [38], and willow trees [29] in Iran. Our study improved the knowledge on the occurrence of fungal trunk pathogens on *Citrus* species showing a decline in symptoms. Further investigations are needed throughout the citrus orchards to determine the potential impact of these fungi on citrus decline.

## 4. Materials and Methods

### 4.1. Tree Sampling and Fungal Isolation

**Figure 5:**
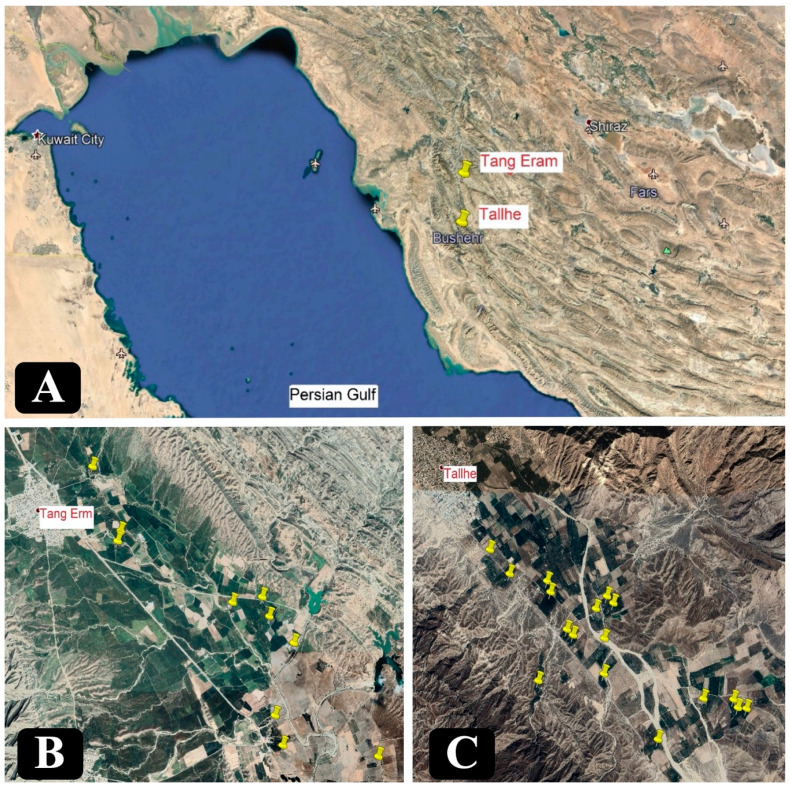
(**A**) Location of the Bushehr province. Two Citrus growing regions were surveyed, Tallhe and Tang Eram. (**B**) Location of surveyed orchards in the Tallhe region. (**C**) Location of surveyed orchards in the Tang Eram region.

During 2014 and 2015, several field surveys were performed in important citrus-producing regions of Bushehr province, Tallhe and Tang Eram. This province is located in the south of Iran, within 28.7621° N latitude and 51.5150° E longitude. Symptomatic wood samples were collected from various species of citrus trees including, acid lime, sweet orange, mandarin, sour orange, sweet lemon (*C. limetta*), and lemon showing yellowing, defoliation, canker, dieback, and gummosis. In total, 325 wood samples were collected from branches of 106 symptomatic trees (15- to 35-year-old) in 27 orchards. A map with the point locations of the sampled orchards is shown in Figure 5. Collected samples were brought to the laboratory and inspected for internal wood lesions and fungal isolation. Small fragments (4 × 4 mm) of symptomatic wood tissues were cut from the edges of wood lesions, surface-sterilized in sodium hypochlorite solution (1.5%) for 60 s, and rinsed three times in sterilized water. Wood chips were dried in sterilized filter paper and placed on PDA amended with 90 to 100 mg/L streptomycin sulfate (PDAS). For each branch sampled, three to five Petri dishes were obtained. All Petri dishes were incubated at 25 °C until fungal colonies were observed. Pure cultures of the fungal isolates were obtained by hyphal-tipping or transferring single conidia to fresh PDA.

### 4.2. Morphological Identification

All fungal isolates were identified initially to the genus level based on colony morphology and main microscopic structures using published articles and descriptions. Botryosphaeriaceae isolates were identified based on colony appearance and conidial morphology [16,108]. To induce sporulation, three to five mycelial plugs from each isolate were placed on 2% water agar (WA; Biokar-Diagnostics) plates amended with sterilized pine needles and incubated at 25 °C under near-ultraviolet light for 15–45 days [42]. Conidial characteristics (size, shape, color, and presence or absence of septa) were recorded for all isolates. *Phaeoacremonium* isolates were grouped based on colony appearance, pigment production on MEA, PDA and oatmeal agar (OA; 60 g oatmeal; 12.5 g agar; Difco, France) and the main microscopic structures (phialide shape and type, conidiophore morphology, size of hyphal warts, and conidial shape and size) [41,57,109]. Identification of *Cadophora* isolates was based on the colony and micro-morphological structures, such as conidiogenous cell size and shape, and conidia. The remaining fungal isolates were identified based on available identification keys and published papers [91,110,111,112,113,114].

### 4.3. DNA Extraction, Amplification, and Sequencing

Identities of representative isolates were confirmed using molecular data. Fungi selected for molecular studies were grown on PDA for 10 to 15 days at 25 °C in the dark. DNA was extracted using an AccuPrep^®^Genomic DNA Extraction Kit (Bioneer, South Korea) following the instructions of the manufacturer. Four primer sets, ITS1/ITS4 [115], EF1-728F/EF1-986R [116], T1/Bt2b [117,118], and ACT-512F/ACT-783R [116] were used to amplify the ITS region ITS1-5.8S-ITS2, portions of the *tef1-α*, BT and ACT genes, respectively. The identification of Botryosphaeriaceae isolates was confirmed by the sequencing of ITS and a partial sequence of *tef-1a*. For *Phaeoacremonium* isolates, a partial sequence of BT and ACT genes were amplified and sequenced. Molecular identifications of other isolates were confirmed by sequence analysis of ITS (*Cadophora*, *Colletotrichum*, *Peyronellea*, *Stilbocrea*, and *Biscogniauxia* isolates), BT (*Colletotrichum* and *Peyronellea* isolates), or *tef1-α* (*Stilbocrea* isolates). The polymerase chain reaction (PCR) was performed in a Techne TC-312 Thermal Cycler (Techne, Cambridge, UK), as described by Hashemi and Mohammadi [29]. For each isolate, 3–4 μL of PCR product was separated by electrophoresis on a 1% agarose gel (UltraPureTM Agarose, Invitrogen) containing ethidium bromide and visualized under UV illumination. The size of the products was evaluated using a 100 bp ladder (Gene Ruler, TMDNA Ladder Mix, Fermentas). PCR products were submitted to Bioneer Corporation (Daejeon, South Korea) for sequencing. MegaBLAST approach of the NCBI database (https://www.ncbi.nlm.nih.gov/) was initially used to identify fungal species.

### 4.4. Phylogenetic Analysis

Due to the broad range of *Phaeoacremonium* spp. obtained in this study, a phylogenetic analysis was carried out for the *Phaeoacremonium* spp. isolates. Sequences from citrus in Iran were aligned with sequences available in GenBank/NCBI. These were compared using MAFFT sequence alignment program v. 6 [119] with ex-type specimens from different hosts. Alignments were inspected in Sequence Alignment Editor v. 2.0a11 [120]. PAUP version 4.0 b 10 [121] was used to perform a partition homogeneity test. The congruence between the ACT and BT datasets was tested at 1000 replicates, and the maximum likelihood (ML) was carried out on the concatenated alignment. The MEGA version 7 software [122] was used for ML analysis. Bayesian information criterion in jModelTest 2.1.10 [123] was used to estimate the best fit model. Single and concatenated datasets were tested for branch support (1000 bootstrap replicates). We included sequences published by Spies et al. as reference sequences [58]. *Pleurostoma richardsiae* CBS 270.33 was included as an outgroup. *Phaeoacremonium* sequences obtained in this study were submitted to GenBank/NCBI (Table 2) and the sequence alignments were deposited in TreeBASE under study number 26006 (http://treebase.org).

### 4.5. Pathogenicity Tests

Pathogenicity tests were carried out with 12 species on detached shoots of *C. aurantifolia* under controlled conditions. These include *Do. viticola, P. italicum*, *P. minimum*, *P. rubrigenum*, and *P. parasiticum* isolated from *C. aurantifolia*, *L. theobromae*, and *Col. gloeosporioides* obtained from *C. sinensis*, *C. luteo-olivacea*, and *N. hyalinum* recovered from *C. limetta*, *P. fraxinopensylvanicum* from *C. limon*, *P. iranianum* from *C. reticulata* and *S. walteri* isolated from *C. aurantium*. The shoots (38–40 cm in length and 2–2.5 cm in diameter) were surface-disinfected with alcohol (96%) and then were wounded at the uppermost internode with a 4-mm cork borer. To assess pathogenicity, wounds were inoculated with a 4-mm colonized PDA agar from 14-days-old cultures. All inoculated sites first were covered by moist cotton and then were wrapped with a strip of Parafilm (Pechiney Plastic Packaging, Menasha, USA). Six shoots per fungal isolate were used, and an equal number of shoots were also inoculated with 4-mm non-colonized PDA agar plugs for negative controls. Inoculated shoots were arranged at random, including the six inoculated shoots per isolate. Inoculated shoots were placed in moist chambers and incubated at 25 ºC. The total, upward, and downward lesion length data were evaluated individually, 40 days after inoculation. Recorded data were checked for normality of distribution by means of the Shapiro–Wilk and Kolmogorov–Smirnov tests. The data were subjected to analysis of variance (one-way ANOVA) using SAS v 9.1 (SAS Institute, Cary, NC, USA) (Dataset S1; Dataset S2). The least significant difference (LSD) test was used for comparison of treatment means at *p* < 0.05. Fungal re-isolations were made from the edges of the lesions on the test and control shoots and placed on PDA. The identity of the re-isolated fungi was confirmed based on morphological characteristics and molecular analysis in order to complete Koch’s postulates. The pathogenicity of other species was not tested in this work because they were identified after the pathogenicity trials had begun on the detached shoots of *C. aurantifolia*.

## Figures and Tables

**Table 1:** Fungal species isolated from *Citrus* species in Iran.

Fungal Species	*Citrus* Species						Total Isolates
	*C. sinensis*	*C. aurantifolia*	*C. reticulata*	*C. limetta*	*C. aurantium*	*C. limon*	
*Phaeoacremonium parasiticum*	1	11	0	0	0	0	12
*P. rubrigenum*	0	4	0	0	0	0	4
*P. minimum*	0	8	0	0	0	0	8
*P. italicum*	0	5	0	0	0	0	5
*P. croatiense*	2	0	0	2	0	0	4
*P. iranianum*	0	0	2	0	0	0	2
*P. fraxinopennsylvanicum*	0	0	0	0	1	1	2
*Phaeoacremonium* sp.	0	2	0	0	0	0	2
*Cadophora luteo-olivacea*	0	0	6	6	0	0	12
*Biscogniauxia mediterranea*	2	0	0	0	0	0	2
*Neoscytalidium hyalinum*	0	7	0	7	0	0	14
*Dothiorella viticola*	7	7	0	0	6	0	20
*Lasiodiplodia theobromae*	7	8	0	0	0	0	15
*Colletotrichum gleoesporioides*	8	0	0	10	0	0	18
*Colletotrichum boninense*	0	0	0	7	0	0	7
*Peyronellea pinodella*	5	6	0	0	0	0	11
*Phoma herbarum*	0	6	0	5	0	0	11
*Phoma fungicola*	5	0	5	4	0	0	14
*Microsphaeropsis olivacea*	6	0	0	0	0	0	6
*Stilbocrea walteri*	0	3	0	0	1	4	8
*Pestalotiopsis* sp.	4	0	0	0	0	0	4
*Fusarium* spp.	2	3	0	1	1	5	12
*Paecilomyces* spp.	2	3	5	1	3	1	15
*Phoma* spp.	3	0	0	0	0	0	3
*Penicillium* spp.	7	4	9	0	4	5	29
*Aspergillus* spp.	11	10	8	8	3	8	48
*Trichoderma* spp.	5	0	2	5	0	1	13
*Alternaria* spp.	3	7	3	10	0	2	25
Total fungal isolates	80	94	40	66	19	27	326
Total number of trees surveyed	39	57	19	43	18	12	188

**Table 2:** Host, origin, and GenBank accession numbers of *Phaeoacremonium* isolates obtained from *Citrus* spp. in Iran (used in phylogenetic studies).

Fungal Isolates		*Citrus* spp.	GenBank Accession Number	
*Phaeoacremonium* Species	Code		b-Tubulin	Actin
*P. parasiticum*	IRNHM-KPH35*	*C. aurantifolia*	KU737504	MT127573
	IRNHM-KPH35E1	*C. sinensis*	MT122909	MT127574
	IRNHM-KPH35E2	*C. aurantifolia*	MT122910	MT127575
	IRNHM-KPH35E3	*C. aurantifolia*	MT122911	MT127576
	IRNHM-KPH35E4	*C. aurantifolia*	MT122912	MT127577
	IRNHM-KPH35E5	*C. aurantifolia*	MT122913	MT127578
	RNHM-KPH35E6	*C. aurantifolia*	MT122914	MT127579
	IRNHM-KPH35E7	*C. aurantifolia*	MT122915	MT127580
	IRNHM-KPH35E8	*C. aurantifolia*	MT122916	MT127581
	IRNHM-KPH35E9	*C. aurantifolia*	MT122917	MT127582
	IRNHM-KPH35E10	*C. aurantifolia*	MT122918	MT127583
	IRNHM-KPH35E11	*C. aurantifolia*	MT122919	MT127584
*Phaeoacremonium* sp.	IRNHM-KPH61	*C. aurantifolia*	KU737517	MT127585
	IRNHM-KPH61E4	*C. aurantifolia*	MT122920	MT127586
*P. italicum*	IRNHM-KPH61E1	*C. aurantifolia*	MT122921	MT127587
	IRNHM-KPH61E2*	*C. aurantifolia*	MT122922	MT127588
	IRNHM-KPH61E3	*C. aurantifolia*	MT122923	MT127589
	IRNHM-KPH63E1	*C. aurantifolia*	MT122924	MT127590
	IRNHM-KPH63E2	*C. aurantifolia*	MT122925	MT127591
*P. rubrigenum*	IRNHM-KPH91E1	*C. aurantifolia*	MT122926	MT127592
	IRNHM-KPH91E2	*C. aurantifolia*	MT122927	MT127593
	IRNHM-KPH91E3	*C. aurantifolia*	MT122928	MT127594
	IRNHM-KPH91E4*	*C. aurantifolia*	MT122929	MT127595
	IRNHM-KPH424E1	*C. aurantifolia*	MT122930	MT127596
	IRNHM-KPH424E2	*C. aurantifolia*	MT122931	MT127597
*P. minimum*	IRNHM-KPH425E1*	*C. aurantifolia*	MT122932	MT127598
	IRNHM-KPH425E2	*C. aurantifolia*	MT122933	MT127599
	IRNHM-KPH425E3	*C. aurantifolia*	MT122934	MT127600
	IRNHM-KPH426E1	*C. aurantifolia*	MT122935	MT127601
	IRNHM-KPH428E1	*C. aurantifolia*	MT122936	MT127602
	IRNHM-KPH430E1	*C. aurantifolia*	MT122937	MT127603
*P. iranianum*	IRNHM-KZ38E1	*C. reticulata*	MT122938	MT127604
	IRNHM-KZ38E2*	*C. reticulata*	MT122939	MT127605
*P. croatiense*	IRNHM-KPH24E	*C. sinensis*	MT122940	MT127606
	IRNHM-KZ40E1	*C. limetta*	MT122941	MT127607
	IRNHM-KZ54E1	*C. limetta*	MT122942	MT127608
	IRNHM-KZ63E1	*C. sinensis*	MT122943	MT127609
*P. fraxinopennsylvanicum*	IRNHM-KZ73E1*	*C. limon*	MT122944	MT127610
	IRNHM-KZ73E2	*C. aurantium*	MT122945	MT127611

Isolates used for pathogenicity tests on detached shoots of *C. aurantifolia*.

## Supplementary Materials

The following are available online at https://www.mdpi.com/2223-7747/9/6/754/s1, Dataset S1: SAS code, Dataset S2: Lesion length data.
