# Supplementary material for: A Survey of Trunk Disease Pathogens within Citrus Trees in Iran
Source: Plants (Basel). 2020 Jun 16;9(6):754. doi: 10.3390/plants9060754 (PMC7355864; doi:10.3390/plants9060754)
Supplement: Supplementary file 1 [file plants-09-00754-s001.zip › Dataset S1.docx]

data read;

infile "C:\sas\data\citrus.csv" dlm=',' firstobs=2;

input method $ grade;

run;

proc univariate data=read normal;

qqplot grade /Normal(mu=est sigma=est color=red l=1);

by method;

run;

title1 'Pathogenicity trials Citrus spp.';

data Citrus;

input Fungi $ Lesion @@;

datalines;

ods graphics on;

proc anova data = Citrus;

class Fungi;

model Lesion = Fungi;

means Fungi / LSD;

run;
